# Quantitative Comparison of Color-Coded Parametric Imaging Technologies Based on Digital Subtraction and Digital Variance Angiography: A Retrospective Observational Study

**DOI:** 10.3390/jimaging10100260

**Published:** 2024-10-18

**Authors:** István Góg, Péter Sótonyi, Balázs Nemes, János P. Kiss, Krisztián Szigeti, Szabolcs Osváth, Marcell Gyánó

**Affiliations:** 1 Department of Vascular and Endovascular Surgery, Heart and Vascular Center, Semmelweis University, Városmajor utca 68, 1122 Budapest, Hungary; gogistvan@gmail.com (I.G.); sotonyi.peter1@semmelweis.hu (P.S.); 2 Kinepict Health Ltd., Szilágyi Erzsébet fasor 31, 1027 Budapest, Hungary; janos.kiss@kinepict.com (J.P.K.); szabolcs.osvath@kinepict.com (S.O.); 3 Department of Interventional Radiology, Heart and Vascular Center, Semmelweis University, Városmajor utca 68, 1122 Budapest, Hungary; nemes.balazs@semmelweis.hu; 4 Department of Biophysics and Radiation Biology, Semmelweis University, Tűzoltó u. 37-47, 1094 Budapest, Hungary; szigeti.krisztian@semmelweis.hu

**Keywords:** color-coded parametric imaging, digital subtraction angiography, digital variance angiography, time–density curve, peripheral artery disease, critical limb ischemia

## Abstract

The evaluation of hemodynamic conditions in critical limb-threatening ischemia (CLTI) patients is inevitable in endovascular interventions. In this study, the performance of color-coded digital subtraction angiography (ccDSA) and the recently developed color-coded digital variance angiography (ccDVA) was compared in the assessment of key time parameters in lower extremity interventions. The observational study included 19 CLTI patients who underwent peripheral vascular intervention at our institution in 2020. Pre- and post-dilatational images were retrospectively processed and analyzed by a commercially available ccDSA software (Kinepict Medical Imaging Tool 6.0.3; Kinepict Health Ltd., Budapest, Hungary) and by the recently developed ccDVA technology. Two protocols were applied using both a 4 and 7.5 frames per second acquisition rate. Time-to-peak (TTP) parameters were determined in four pre- and poststenotic regions of interest (ROI), and ccDVA values were compared to ccDSA read-outs. The ccDVA technology provided practically the same TTP values as ccDSA (r = 0.99, R^2^ = 0.98, *p* < 0.0001). The correlation was extremely high independently of the applied protocol or the position of ROI; the r value was 0.99 (R^2^ = 0.98, *p* < 0.0001) in all groups. A similar correlation was observed in the change in passage time (r = 0.98, R^2^ = 0.96, *p* < 0.0001). The color-coded DVA technology can reproduce the same hemodynamic data as a commercially available DSA-based software; therefore, it has the potential to be an alternative decision-supporting tool in catheter labs.

## 1. Introduction

Minimally invasive endovascular interventions play an increasingly important role in the treatment of cardiovascular disorders and certain types of benign or malignant oncological states [1]. These procedures require a detailed visualization of blood vessels during the intervention. The reference standard method for this purpose is digital subtraction angiography (DSA), which subtracts a mask image from the subsequent contrast agent-enhanced image series, thereby allowing it to show the vasculature in the site of intervention without other disturbing anatomical structures [2].

The morphological analysis of key lesions can be performed by measuring the degree of stenosis; however, the visual evaluation of gray-scale DSA images gives two-dimensional projected information of a three-dimensional structure, which can lead to misdiagnosis by evaluating a stenotic vascular segment as normal. Without a quantitative evaluation of hemodynamic conditions, the intraprocedural assessment of stenotic lesions can be subjective. In response to this medical need, a new quantitative technology, called color-coded parametric angiography (also termed as color-coded DSA, quantitative DSA, or two-dimensional perfusion angiography), has been developed [3,4,5,6]. The method creates a time–density (also called time–attenuation) curve and extracts time- and attenuation-related parameters from DSA acquisition. These parameters are used to create a color-coded image, where the colors represent the arrival time of the contrast agent at any point of the angiogram (the earliest is red, the latest is blue) and the brightness is proportional to the attenuation. Thus, this single image contains quantifiable spatiotemporal information on the blood flow and visualizes hemodynamic conditions in the target area.

Color-coded parametric imaging has been tested in a number of clinical studies over the last 15 years [5,6,7,8,9,10,11,12,13,14,15,16,17,18]. Certain ccDSA parameters showed strong correlation with the clinical outcome in stroke treatment [7,8] and helped the evaluation of carotid cavernous fistulas better than gray-scale DSA videos [9]. In comparison with DSA videos, color-coded images significantly improved diagnosis and treatment planning in cerebrovascular disorders, and the positive effect was greater for less experienced readers [5]. Parametric imaging proved to be useful in other endovascular procedures as well, including lower limb interventions [10,11], the intraprocedural evaluation of type B aorta dissections in thoracic endovascular aortic repair [12], the identification of bleeding points [13], the intraprocedural evaluation of genicular embolization [14], spleen embolization [15], the evaluation of hemodynamic changes during the endovascular treatment of brain arteriovenous malformations [16], and the prediction of brain aneurysm occlusion after embolization [17,18]. In spite of these promising results, the ccDSA technology did not become an everyday tool because of certain disadvantages (e.g., the high radiation load during ccDSA acquisitions).

Digital variance angiography (DVA) is a recently developed image processing alternative to DSA. The technology is based on the principles of kinetic imaging [19]. In contrast to DSA, DVA does not use a mask for subtraction. Instead, DVA calculates the standard deviation of changing pixel intensities for each pixel, which generates a standard deviation map, the so-called DVA image. The motion of the contrast agent creates high standard deviation values, while the stationary background and the background noise give low standard deviation values, resulting in high contrast and a greatly improved image quality. This quality advantage has been validated in lower limb interventions [20,21,22,23], carotid angiography [24], prostatic artery embolization [25], and the transarterial chemoembolization of liver tumors [26]. 

The quality reserve of DVA also provides opportunity for dose management [24,27,28]. As the technology can generate color-coded DVA (ccDVA) images, our hypothesis was that ccDVA could solve the problem of a high radiation dose of ccDSA, provided it can reliably reproduce the parametric data generated by ccDSA. This assumption is not evident as the algorithms of DSA and DVA are completely different. Our aim, therefore, was to compare the key time-related parameter, the time to peak (TTP), obtained with DVA, to the TTP values of a commercially available color-coded DSA software in the lower limb intervention of patients with critical limb-threatening ischemia (CLTI) in order to provide evidence for the validity of ccDVA measurements.

## 2. Materials and Methods

### 2.1. Patients

Our retrospective observational study prospectively enrolled 19 CLTI patients (mean ± SD age 68.6 ± 8.0 years, 42% male) (Fontaine stage III–IV) who underwent peripheral vascular intervention at the Heart and Vascular Center, Semmelweis University, between September and December of 2020. The detailed demographic data are shown in Table 1. All patients signed an informed consent and permitted the use of their anonymous acquisitions for research purposes. There was no change in the interventional protocol recommended for color-coded imaging, and the standard of care was given to all subjects.

### 2.2. Image Acquisition

Each procedure was performed from either a brachial or femoral access using the Seldinger technique and locoregional anesthesia. Image acquisition was made on a Siemens Artis Zee system (Siemens Healthineers, Erlangen, Germany), which included the parametric angiography software iFlow (Siemens Healthineers, Erlangen, Germany) with a Medrad Avanta automated injector (Bayer, Berlin, Germany). During the procedure, a pre-interventional angiogram was created by injecting 3–8 mL of iodinated contrast media (Ultravist 370, Bayer) at a 2–6 mL/s flowrate through a single endhole catheter placed selectively into the target artery, which was the superficial femoral artery in 45% (*n* = 10), the popliteal artery in 27% (*n* = 6), the common or external iliac artery in 18% (*n* = 4), and the crural branches in 9% (*n* = 2) of all cases (*n* = 22). Image acquisition occurred at 4 or 7.5 frames per second (FPS). These higher rates are recommended for the generation of good quality color-coded images by the iFlow software (Syngo Workplace version VD11B, Siemens, Germany). The treatment of stenoses was conducted by “plain-old-balloon-angioplasty (POBA)” and/or by the placement of a self-expanding stent into the artery. Three patients had multiple level lesions, and they were treated in two sessions. These interventions were handled as separate cases. 

### 2.3. Image Processing

Pre- and postinterventional color-coded images were generated retrospectively. The ccDSA images were prepared from the DSA acquisitions by the iFlow software package running on a Syngo workstation (XVP VD11B and VD11C; Siemens Healthineers, Erlangen, Germany), whereas the ccDVA images were calculated from the unsubtracted raw acquisition by the Kinepict Medical Imaging Tool software (KMIT 6.0.3; Kinepict Health, Budapest, Hungary) running on a separate computer. Each workstation allowed further postprocessing, such as pixel shift for motion correction and setting the brightness and contrast. 

### 2.4. Data Collection

**Figure 1:**
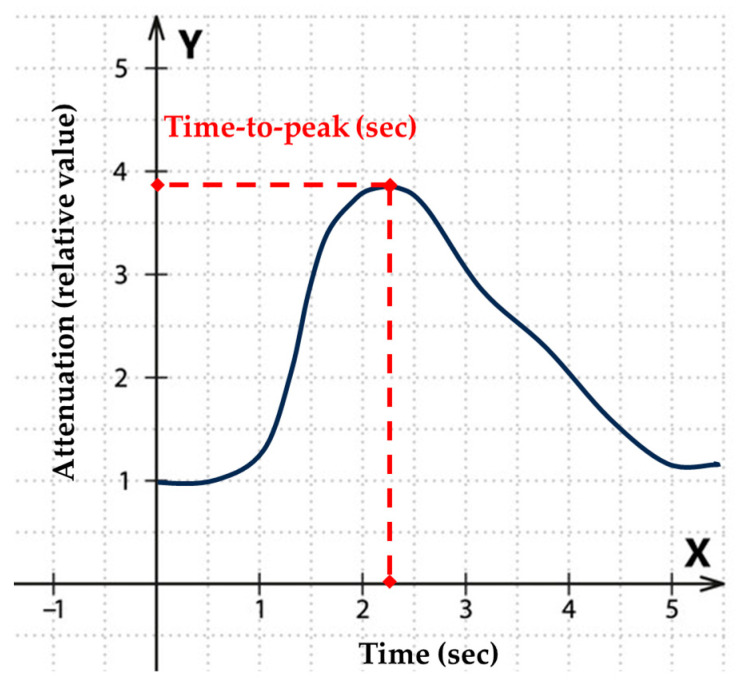
Calculation of the time-to-peak (TTP) parameter from the time–attenuation curve. The red dotted lines indicate the level of peak attenuation (vertical line) and the time at the peak (horizontal line).

Each software allows the placement of regions of interests (ROIs), for which they can generate time–attenuation curves. These curves can be used to calculate the time-to-peak (TTP) parameter, which gives the time from the start of acquisition until the development of the maximum average contrast intensity in the given ROI (Figure 1). 

**Figure 2:**
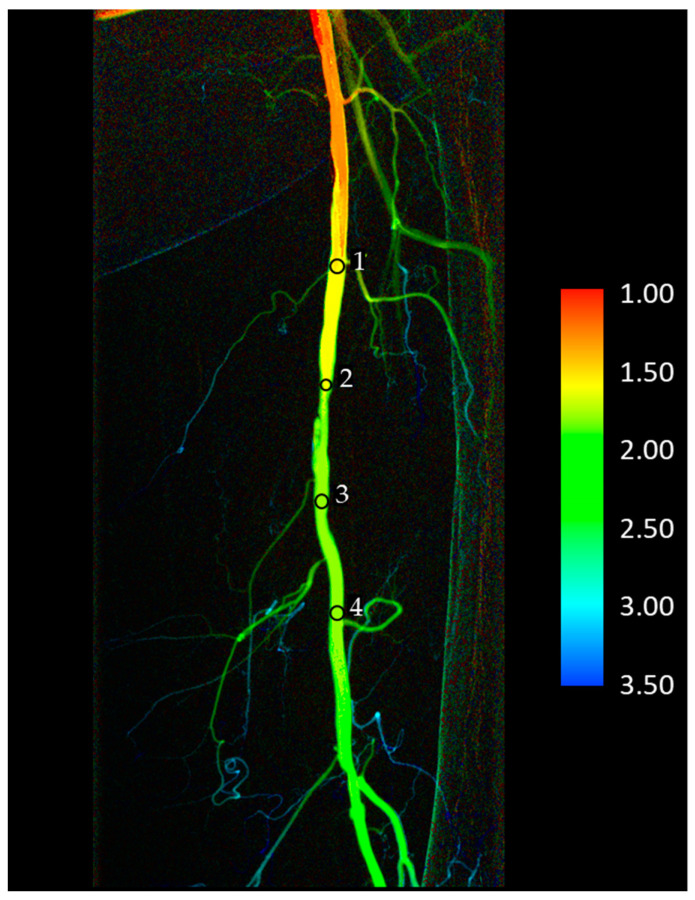
Typical placement of region of interests (ROIs) on a color-coded DVA image. The color bar shows the connection between the colors and the elapsed time (in seconds) measured from the injection of contrast media. In three cases (iliac and talocrural lesions), there was no space to place the fourth ROI. The ‘change in passage time’ calculations did not include these patients. The numbers besides the round shaped selections (ROIs) are serial numbers, indicating the sequence of ROI placement.

Usually, 4 ROIs were placed on the images: the first being 5–10 cm proximally from the stenotic lesion; the second being at the beginning of the lesion; the third being at the end of the lesion; and the fourth being 5–10 cm distally from the lesion. (Figure 2). In three cases, the fourth ROI could not be placed due to anatomical reasons. Only the ROI1-ROI3 data were included in calculations for these patients, and they were excluded from passage time calculations (see below). The same ROI sets were used on the pre- and postinterventional images. 

As our aim was to compare the performance of KMIT to iFlow, the placement of ROIs on DSA images defined the position of ROIs on DVA images. The analogous ROIs were placed at identical positions with identical sizes, with the help of an open-source mouse-position tracking software (MPos, version 0.5, Bluegrams, https://github.com/Bluegrams, accessed on 13 October 2024).

Passage time was defined as the difference between TTPROI4 and TTPROI1, i.e., the time necessary for the bolus to flow across the target area. The change in passage time is the difference between the passage time before and after intervention. This parameter is an indicator of treatment efficacy, and normally, a positive number being used as the passage should be accelerated due to angioplasty/stenting. 

### 2.5. Data Analysis

TTP data were separately analyzed according to the acquisition protocol and the ROI position. The change in passage time was also compared between the two parametric imaging technologies. The TTP and passage time parameters calculated by iFlow and KMIT were compared using the Pearson correlation test. SPSS (version 28, IBM Corp Armonk, NY, USA) and Prism 8.4 (GraphPad, San Diego, CA, USA) were used for statistical analysis. 

## 3. Results

Altogether twenty-two pre-interventional and twenty-two postinterventional acquisitions were processed from nineteen patients, as two independent interventions were included from three patients. Two different acquisition protocols (4 FPS and 7.5 FPS) and four ROI positions (ROI1-4) were tested, except for in three patients, where only three ROI positions (ROI1-3) could be placed. All these patients belonged to the 4 FPS group. When the change in passage time was investigated, only those patients who had four ROIs were included.

### 3.1. Correlation of TTP Parameters in Different Acquisition Protocols

**Figure 3:**
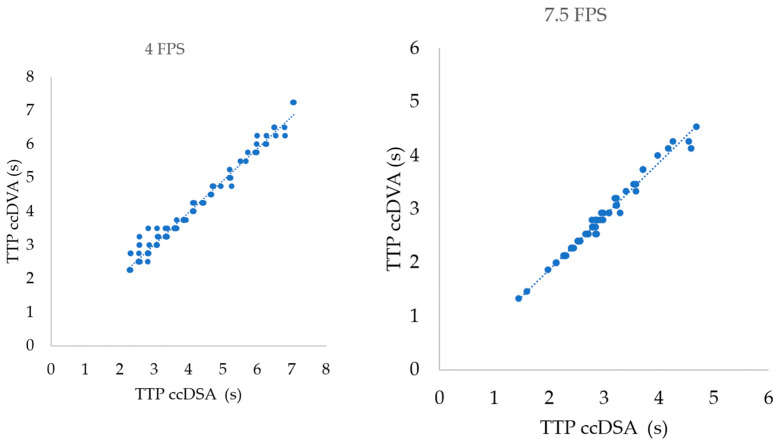
Correlation of TTP values calculated by ccDSA and ccDVA in different acquisition protocols. Left panel: correlation of 106 ROI pairs in 4 FPS acquisitions. Right panel: correlation of 64 ROI pairs in 7.5 FPS acquisitions.

The correlation was not dependent on the acquisition protocol and was extremely high in all cases (Figure 3). The Pearson correlation coefficient (r) was 0.99 (*p* < 0.0001) and the R^2^ was 0.98 in both the 4 FPS and the 7.5 FPS groups (Table 2).

### 3.2. Correlation of TTP Parameters in Different ROI Positions

**Figure 4:**
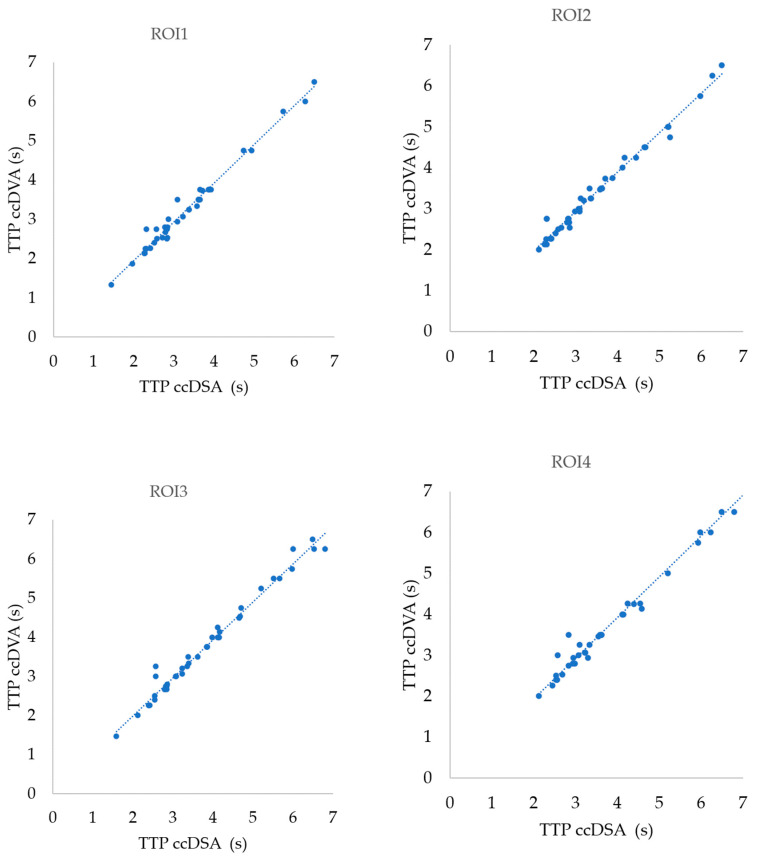
Correlation of TTP values calculated by ccDSA and ccDVA in different ROI positions. In all cases, 44 ROI pairs were included in the analysis except for ROI4, where only 38 ROI pairs were used.

The correlation was not dependent on the ROI position and was extremely high in all cases (Figure 4). The Pearson correlation coefficient (r) was 0.99 (*p* < 0.0001) and the R^2^ was 0.98 in all ROI positions (Table 2). 

### 3.3. Correlation of Change in Passage Time Following the Intervention

**Figure 5:**
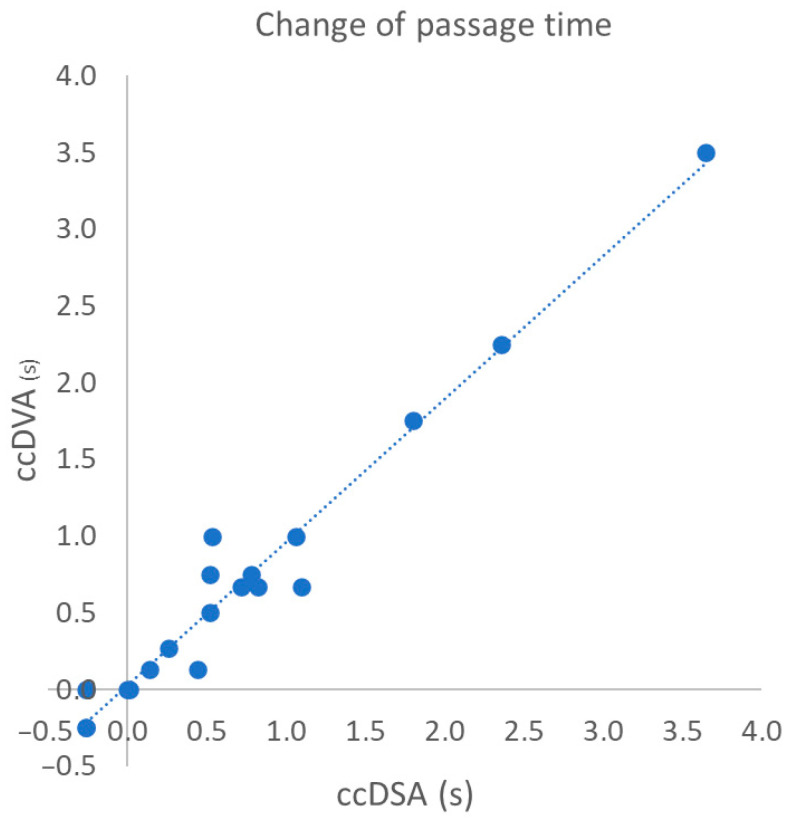
Correlation of the change in passage time calculated from TTP data of ccDSA and ccDVA. We could not place the 4th ROI in 3 interventions. Therefore, only 19 ROI pairs were used in the analysis. The change in passage time was calculated as the differences in (TTP_ROI4_-TTP_ROI1_) before and after intervention.

The passage time (for more details, see the ‘Material and Methods’ section) is an important parameter which characterizes the hemodynamic conditions of in the target area. Beyond the raw TTP data, our aim was to test the changes in this functional parameter that can reflect the efficacy of treatment. The change in passage time was detected very similarly by both technologies; the correlation was extremely high (Figure 5). The Pearson correlation coefficient (r) was 0.98 (*p* < 0.0001) and the R^2^ was 0.96 (Table 2). 

## 4. Discussion

Our aim was to investigate the reliability of ccDVA to determine time-related parameters in lower limb interventions aiming to restore or improve the circulation in lower extremity arteries of CLTI patients. For this purpose, we compared the TTP values obtained by ccDVA to those obtained by a commercially available ccDSA software. The comparison was justified as the two technologies use different algorithms. The ccDSA software extracts data from the subtracted DSA acquisition, while the ccDVA software calculates parametric data from the raw unsubtracted series. The correlation analysis clearly shows that ccDVA reproduces the TTP parameters of the ccDSA software with high fidelity, and the performance is not dependent on the applied frame rate or the position of the ROIs. Another time-based parameter, the change in the passage time was also compared as this value might help to evaluate the efficacy of intervention. Again, the DVA-based software gave practically the same values as the DSA-based solution, indicating that the time parameters obtained with ccDVA are valid and can be used in clinical practice similarly to the commercially available comparator technology. 

The principles of color-coded angiographic imaging emerged four decades ago [3,4], but the major angiography manufacturers introduced their own color-coded solutions only in the last 10–15 years. Although clinical studies indicated the potential usefulness of this technology [7,8,9,10,11,12,13,14,15,16,17,18], color-coded parametric imaging is not very widespread. There are several factors that make daily use difficult. It is essential to define strict clinical protocols for this type of imaging for the sake of reproducibility and for valid, clinically useful measures. Besides image acquisition parameters, other circumstances should be considered, such as fixing the patient’s leg with special equipment (footrest) to minimize motion artifacts, which may distort results and may not be eliminated by image registration algorithms. Nevertheless, perhaps the most severe problem is that good quality color-coded images require repeated longer acquisitions with higher frame rates (4 FPS and above) in order to obtain good time resolution and a clear picture on the hemodynamic conditions. This is a very significant radiation burden for the patients and for the medical staff as well. 

Clinical significance. The recently developed DVA technology provides higher CNR and better image quality than DSA [20,21,22,23,24,25,26], and this quality advantage can be used for dose management. DVA allowed for the reduction in the dose/frame parameter by 70%, which resulted in a more than 60% reduction in the DSA-related DAP values without compromising the image quality of angiograms [27,28]. As the ccDVA technology proved to be a reliable substitute for the currently available DSA-based parametric imaging, it could solve the problem of high radiation burdens through its dose management capability and could help in the wide-spread use of color-coded parametric imaging.

Limitations. The study involved a relatively low number of patients. Nevertheless, the larger number of measurement points increased the power of calculation and was sufficient to reach a solid conclusion. Another limitation is that we have tested only the time-related parameters. Both software can also calculate attenuation-related parameters, like the area under curve or the peak attenuation. Nevertheless, these parameters have arbitrary units and are less indicative when small ROIs are used (like in our case), which includes only a blood vessel cross section. The reliability of these parameters could be investigated in perfusion-type interventions, where the judgment of tissue blush or the arteriovenous phase is more important. Based on the current positive results, we plan to perform such studies in the field of prostatic artery embolization, uterine fibroid embolization, or cerebral imaging after thrombectomy. 

## 5. Conclusions

Our data clearly show that the color-coded DVA technology can reproduce the same time-related parameters as the commercially available DSA-based color-coded parametric imaging; therefore, ccDVA can be a potential alternative in clinical practice. The previously described quality reserve of DVA might allow for a very significant reduction in the radiation dose applied during color-coded imaging, which might help to increase the use and significance of parametric imaging in endovascular intervention. However, the validation of these claims requires further prospective clinical studies.

## Figures and Tables

**Table 1:** Demographic data.

Sex	Number of Patients	Age ^1^ (Years)	BMI ^1^	GFR ^1^ (mL/min/1.73 m^2^)
Male	8	69.6 ± 7.0	25.0 ± 4.5	65.3 ± 26.7
Female	11	67.9 ± 8.8	26.6 ± 4.6	55.8 ± 26.2

^1^ All data are mean ± SD. BMI: body mass index. GFR: glomerular filtration rate.

**Table 2:** Pearson correlation analysis of time-related parameters. TTP: time to peak; ROI: region of interest; *n*: the number of ROI pairs included in the analysis, FPS: frame per second.

Correlation Group (*n*)	Pearson r	95% Confidence Interval	R^2^	Two-Tailed p
TTP 4 FPS (106)	0.9889	0.9837–0.9924	0.9779	<0.0001
TTP 7.5 FPS (64)	0.9917	0.9864–0.9950	0.9835	<0.0001
TTP ROI1 (44)	0.9899	0.9814–0.9845	0.9799	<0.0001
TTP ROI2 (44)	0.9903	0.9822–0.9947	0.9807	<0.0001
TTP ROI3 (44)	0.9902	0.9819–0.9947	0.9804	<0.0001
TTP ROI4 (38)	0.9904	0.9814–0.9950	0.9808	<0.0001
TTP all ROI (170)	0.9905	0.9871–0.9930	0.9811	<0.0001
Δ passage time ^1^ (19)	0.9806	0.9491–0.9927	0.9615	<0.0001

^1^ The change in passage time (Δ) was calculated as the differences in (TTP_ROI4_-TTP_ROI1_) before and after intervention.

## Data Availability

Study protocols, ethical approval documents, fully anonymized images, and TTP read-outs are available on request at the Heart and Vascular Center and at the head office of Kinepict Health Ltd.
